# Consequences of host-microbiome interactions in preterm infants

**DOI:** 10.1128/iai.00501-24

**Published:** 2025-08-11

**Authors:** Isabel Erickson, Jessica Tung, Drew J. Schwartz

**Affiliations:** 1 Department of Pediatrics, Washington University School of Medicine 159758, St. Louis, Missouri, USA; 2 Center for Women’s Infectious Diseases, Washington University School of Medicine 12275 , St. Louis, Missouri, USA; 3 Department of Molecular Microbiology, Washington University School of Medicine 169015, St. Louis, Missouri, USA; 4 Department of Obstetrics & Gynecology, Washington University School of Medicine 169052, St. Louis, Missouri, USA; University of Pittsburgh, Pittsburgh, Pennsylvania, USA

**Keywords:** microbiome, sepsis, bloodstream infection, infant, serious bacterial infections

## Abstract

Preterm infants are highly susceptible to bacterial infections and inflammatory diseases. These vulnerabilities arise from disruptions in gut microbiome structure and function, immune system immaturity, and underdeveloped organ systems. In this review, we explore the role of the gut microbiome in neonatal health. With a specific focus on preterm infants, we outline how microbiome disruption contributes to negative clinical outcomes. First, we provide an overview of infant gut microbiome development, highlighting key factors that influence its trajectory. Next, we examine the interplay between the infant gut microbiome and the development of systemic and intestinal immune systems, with emphasis on how microbiome perturbations related to preterm birth alter host-microbiome interactions and the overall immune landscape. We then discuss the role of altered gut composition in disease states common to preterm infants, such as sepsis, bacterial infections, and necrotizing enterocolitis. Finally, we discuss current and future diagnostics and treatments and offer our perspective on future research to untangle the host-microbiome interface in early life.

## INTRODUCTION

The gut microbiome is composed of bacteria, viruses, and fungi. Its development occurs in parallel with and influences the maturation of the systemic and intestinal immune systems, including by contributing to colonization resistance to invasive pathogens (1–5). In infants born prematurely, progression of gut microbial colonization is disrupted, often with grave consequences. In this review, we focus on how bacterial members of the microbiome contribute to these outcomes.

Due to a combination of host and environmental factors, the preterm gut microbiome is dysbiotic compared to term infants. Here, we define dysbiosis as low species richness, depletion of generally beneficial anaerobes such as *Bifidobacteria*, and domination by potential pathogens (6, 7). These pathogens can translocate across the intestinal epithelium, leading to systemic bacterial infection and sepsis. Additionally, disruptions in the immune-microbiome axis can contribute to preterm infants’ risk for necrotizing enterocolitis (NEC), an inflammatory disease of the gut (8).

Bloodstream infection (BSI) and NEC, with or without accompanying sepsis, are significant causes of mortality and morbidity in preterm infants (9, 10). Therefore, understanding how gut microbiome-immune interactions contribute to negative clinical outcomes is a key research priority. Here, we review current knowledge on infant gut microbiome assembly and its impact on the immune system, with a focus on preterm infants and the role of dysbiosis in bacterial infections, sepsis, and NEC. Finally, we will outline current and future prospects for diagnosis, treatment, and prevention of these adverse outcomes.

## MICROBIOME DEVELOPMENT

### Initial colonization

**Fig 1:**
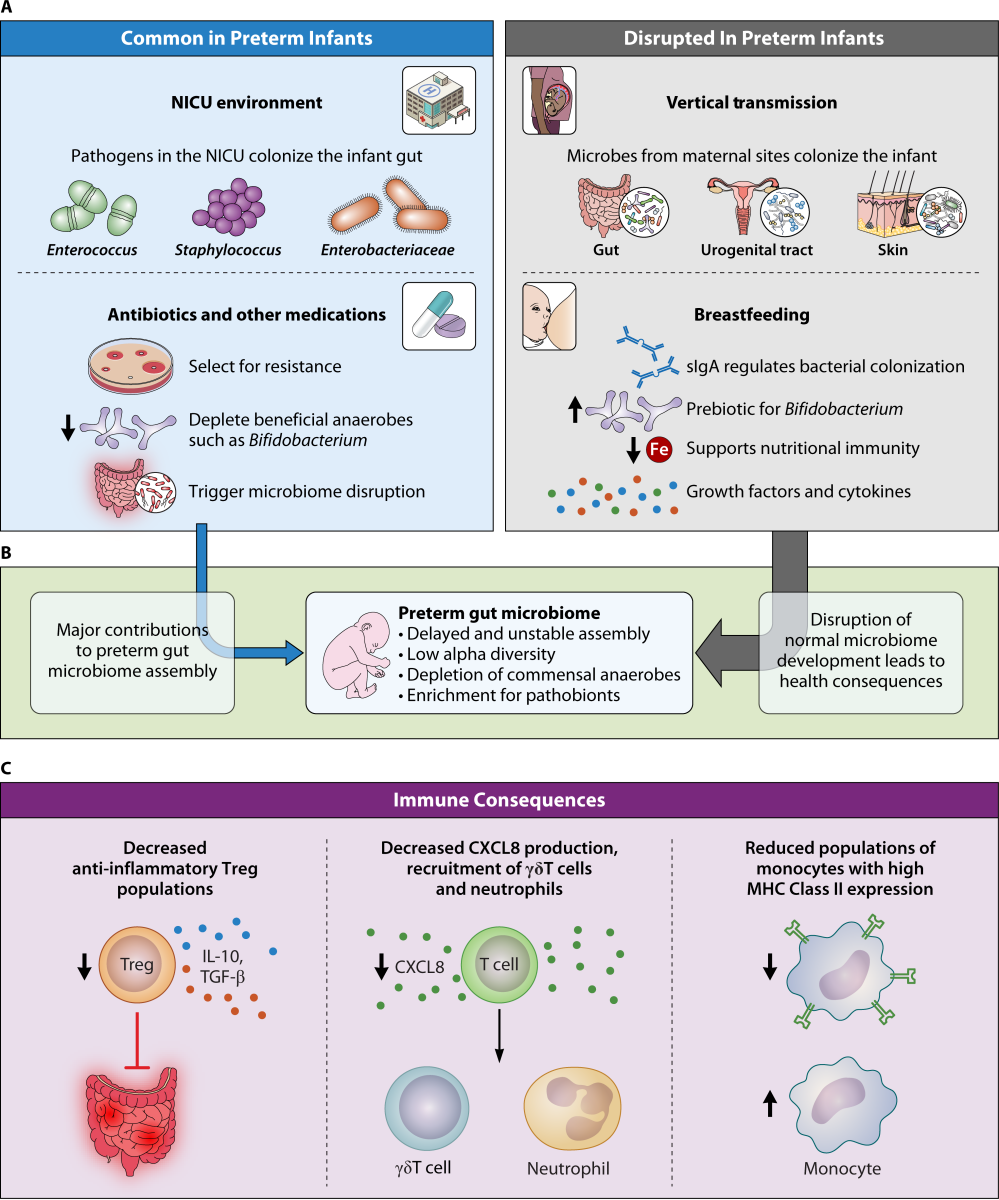
Preterm gut microbiome assembly and immune consequences. (**A**) Exposure to antibiotics and to pathogens in the neonatal intensive care unit (NICU) environment shapes preterm gut microbiome assembly. Vertical transmission and breastfeeding are often disrupted. (**B**) This leads to delayed and unstable microbiome assembly, often dominated by a pathogen with >50% relative abundance. (**C**) Immune outcomes linked to preterm birth or preterm microbiome composition.

Gut microbiome assembly begins at birth. Infants are colonized with microbes from maternal and environmental sources and share strains with their mother’s vaginal and gut communities (Fig. 1A) (11–14). This vertical transmission is influenced by birth mode; vaginally born infants initially have gut microbiomes resembling their mother’s vaginal microbiome, whereas those delivered by Cesarean section have gut microbiomes more similar to the maternal skin microbiome (15). These differences can have long-term consequences; delivery mode affects microbiome composition well into infancy, and birth by C-section is a risk factor for diseases linked to altered gut microbiome composition such as asthma (16, 17).

In preterm infants, several factors—high rates of C-section, intestinal immaturity, hospitalization in neonatal intensive care units (NICUs), antibiotic exposure, and interruptions to breastfeeding—disrupt initial microbiome assembly (Fig. 1A) (6, 7, 18–21). As a result, hospitalized preterm infants have lower abundances of vertically transmitted, beneficial microbes such as *Bifidobacterium* (7). *Bifidobacterium* spp. provide colonization resistance against pathogens (22), and their depletion is associated with intestinal and systemic inflammation in infants. Oral administration of *Bifidobacterium infantis* EVC001 is sufficient to lower inflammatory markers of enteric inflammation (23, 24).

This disruption of physiological microbiota assembly leaves hospitalized infants vulnerable to colonization by bacteria from the NICU environment (Fig. 1A), including common causative agents of BSI, such as *Enterobacteriaceae*, *Staphylococcus* spp., *Streptococcus* spp., and *Enterococcus* spp. (25, 26). Time-resolved 16S rRNA gene sequencing of NICU surfaces and matched infant microbiomes shows these taxa are detected in the environment before the infant gut (27, 28). Bacterial strains, confirmed through precise strain tracking with metagenomic sequencing, can be shared between infants hospitalized simultaneously or months apart, suggesting a shared reservoir of potential pathogens in the NICU environment (28–30). Moreover, similar *Staphylococcus epidermidis* strains have been detected in infants across multiple NICUs in the Midwestern USA, suggesting adaptation to the NICU environment (29). Thus, the NICU environment exerts important influence over the nascent preterm gut microbiome and interrupts healthy development. Investigation into how NICU hospitalization interacts with gestational age (GA), delivery mode, feeding type, and antibiotic treatment to shape preterm infant gut microbiome assembly remains a priority.

### Composition and succession

In the USA and Europe, healthy term infant microbiome development follows a stereotyped succession, where facultative anaerobes, including *Enterobacteriaceae* and *Lactobacillus,* are gradually replaced by obligate anaerobes such as *Bifidobacterium*, *Bacteroides*, and *Clostridium* spp. (1, 19, 31). Initially composed of few taxa, the infant gut microbiome increases in species richness until weaning, when the introduction of solid food drives rapid microbiome changes (31–33). This succession pattern can be influenced by various factors, including delivery mode, pre-weaning diet, antibiotic exposure, and hospitalization (Fig. 1A) (7, 17, 19, 31, 34, 35). As a result, infants have a more variable microbiome composition between individuals and over time relative to adults (36). This reflects changes that occur during typical microbial succession and the infant gut microbiome’s vulnerability to perturbations resulting in dysbiosis.

Gut microbiome composition and succession differs significantly in preterm infants compared to term infants (Fig. 1B). Preterm infants have less diverse microbiomes that mature more slowly, with obligate anaerobes colonizing later in life (7, 37). Initially, the preterm infant gut is dominated by *Staphylococcus* and Bacilli, before transitioning to communities dominated by potential pathogens, including *Klebsiella pneumoniae*, *Enterococcus faecalis*, and *Escherichia coli* (6, 29, 37, 38). Research suggests this transition is driven in part by interactions between *Staphylococcus* and *Klebsiella; Staphylococcus* promotes *Klebsiella* growth, which in turn, inhibits *Staphylococcus* (37). Notably, preterm infants often have simple gut microbiomes dominated by one highly prevalent species with >50% relative abundance (6, 29). While many differences in microbiome maturity between preterm and term infants resolve by 12–15 months of age (7), overall, infancy is a critical window where dysbiosis renders preterm infants vulnerable to bacterial infections, NEC, and impaired immune development.

### Antibiotics and other medications

Antibiotics, such as beta-lactam/aminoglycoside combinations, are frequently prescribed in the NICU, with ampicillin and gentamicin both among the top 5 commonly prescribed drugs in NICUs worldwide (39). This use is partly driven by empiric antibiotic administration upon suspicion of sepsis. Because they are at high risk for sepsis and invasive bacterial infection, most (>80%) extremely low-birth-weight (<1,000 g) and very low-birth-weight (VLBW, <1,500 g) infants receive empiric antibiotics at some point during hospitalization (18, 40).

Antibiotic exposure is linked to delayed microbiota maturation in human infants (19, 41) and mice (42). Additionally, antibiotic treatment increases the abundance of organisms carrying antibiotic resistance genes (ARGs) in the infant gut microbiome (7, 19, 42, 43) while lowering microbiome diversity (Fig. 1A) (19, 20). In term infants with suspected early-onset sepsis, antibiotics have been shown to decrease beneficial *Bifidobacterium* and *Bacteroides* commensals while increasing potentially pathogenic *Enterococcus*, *E. coli*, and *Klebsiella* (20). The impact of antibiotic exposure between 6 weeks and 1 year of life varies across different *Bifidobacterium* and *Bacteroides* spp. (43). These dynamics are relevant due to strain and species-specific functional differences, which exert different effects on microbiome assembly and host health (22, 44). The effect of antibiotics may be modulated by other exposures, including diet and other medications; breastfeeding has been shown to protect against antibiotic-mediated depletion of *Bacteroides* from the infant gut (45).

Antibiotic classes differentially deplete taxa from the gut microbiome, which allows for the introduction or enrichment of other species (20). For example, ampicillin or gentamicin treatment increases *Enterobacteriaceae*, and vancomycin or gentamicin exposure increases *Enterococcaceae* (30). Vancomycin, ampicillin, and gentamicin, the three most commonly used antibiotics in NICUs, increase species richness in some infants and decrease richness in others (6). The direction of the effect of vancomycin and gentamicin can be predicted based on ARG presence and *E. coli* and *Staphylococcus aureus* abundance prior to treatment (6). In contrast, meropenem, cefotaxime, and ticarcillin-clavulanate are consistently associated with reduced species richness (6). In the NICU, administration of glycopeptides, lincosamides, or broad-spectrum antibiotics (such as carbapenems and third- and fourth-generation cephalosporins) can lead to dramatic shifts in microbiome composition; these shifts often introduce *Enterobacter cloacae*, *E. coli*, *E. faecalis*, and *K. pneumoniae* while depleting commensal genera like *Bifidobacterium* and *Veillonella* (29). Shifts in taxonomic composition are also associated with the introduction of ARGs (29). Non-antibiotic medications such as caffeine, oral iron, and steroids also precipitate shifts in microbiome composition (29). *Klebsiella* is the genus most affected by non-antibiotic medication administration (29). Understanding how medications enrich potential pathogens in the gut microbiome may help clinicians anticipate how treatments modulate risk for sepsis and other serious complications.

### Human milk

Human milk is one of the most important exposures that shapes the infant gut microbiome (19, 29, 35), and is considered to be immunomodulatory (46–49), anti-infective (50, 51), and prebiotic (Fig. 1A) (52). These benefits are conferred through a complex array of breast milk components, including human milk oligosaccharides (HMOs), secretory immunoglobulin A (sIgA), antimicrobial proteins, growth factors, and cytokines (53). HMOs act as specific prebiotics for commensal *Bifidobacterium* and *Bacteroides* in the distal small intestine and colon (46, 52, 54–56), while sIgA mediates host-microbe interactions at mucosal surfaces, promoting either exclusion from or association with mucosal sites (57, 58). Additionally, human milk may mediate microbiome development through nutritional immunity, in part by promoting an iron-restricted environment that prevents growth of pathogenic bacteria with high iron requirements (59, 60). Alongside their effects on the gut microbiome, hormones, interleukins, and microRNAs in human milk act directly on the infant gut epithelium, immune system, and metabolism. Together, these components support normal microbiome and immune development, reducing the risk of NEC, respiratory infection, atopic dermatitis, obesity, and allergic disease (61–64).

Despite these benefits, preterm infants are breastfed at lower rates than term infants (65, 66). Preterm infants in the NICU often initially cannot be fed by mouth and instead receive intravenous nutrition or milk via nasogastric tube. This delays the opportunity for direct breastfeeding until approximately 34 weeks of corrected GA, at which point initiating lactation is difficult (67, 68). Due to insufficient milk supply, preterm infants often receive pasteurized and fortified donor milk instead of mother’s own milk (MOM) (69). However, donor milk pasteurization modifies or destroys many bioactive components in human milk and abrogates milk IgA binding to bacteria (70, 71). Reduced exposure to human milk, particularly MOM, may contribute to preterm infants’ increased risk for bacterial infections and NEC.

## IMMUNE DEVELOPMENT

### Preterm disruption

The neonatal immune system is immature and has functional differences from the adult immune system (72, 73). This allows tolerance to foreign antigens encountered at birth and intestinal colonization by commensals but also causes infants to be more vulnerable to infection (73). Because infants have relatively few conventional T cells and limited ability to mount type 1 helper T cell responses (74, 75), activation of the innate immune response (including neutrophils, monocytes, and γδT cells) is key to controlling infection.

Compared to this already vulnerable baseline, preterm infants exhibit altered peripheral blood immune cell and cytokine profiles, which may further contribute to their risk for infection (Fig. 1C) (76–78). For instance, monocyte expression of the major histocompatibility complex (MHC) Class II molecule human leukocyte antigen - DR isotype (HLA-DR), which presents antigens to T cells, is negatively correlated with GA (78–81). Low MHC Class II expression, along with impaired monocyte phagocytic capacity, is associated with infection and sepsis in preterm infants (78–83). Similarly, production of CXCL8, a chemokine that activates γδT cells and neutrophils to combat infection, is lower in preterm infants with low GA or suspected infection (83, 84). Together, these data suggest preterm infants have specific immune disruptions that impair their ability to respond to bacterial infections. In combination with an immature gut barrier that permits increased bacterial translocation (85, 86), these deficiencies place preterm infants at greater risk for BSI and sepsis originating from the gut microbiome.

While it is clear that the gut microbiome plays a role in immune system education, the impact of preterm gut microbiome disruption on immune outcomes is incompletely defined. Studies have found few statistically significant associations between gut microbiome composition and immune cell or cytokine profiles (83, 87). However, preterm infants with low-diversity gut microbiomes dominated by Bacilli or γ-Proteobacteria have more heterogeneous immune profiles than infants with higher gut microbiome diversity, suggesting that gut microbiome dysbiosis may variably disrupt a stereotyped immune developmental trajectory (76).

### Immune tolerance

Microbial exposures during early life promote the development of immune tolerance or prevention of inappropriate immune responses to antigens. Early-life dysbiosis is linked to chronic inflammatory conditions, including asthma, obesity, and Crohn’s disease (88–93). Specific commensal taxa are associated with alterations in cytokine and IgA production, as well as tonic interferon signaling (94–98), induction of immune cell subsets, and increased barrier integrity (99). This suggests that certain commensal species, frequently depleted in preterm infants, are key mediators of proper early-life immune development. Interestingly, preterm infants are at increased risk for asthma and wheeze, although the underlying mechanism is unknown (100).

Induction of regulatory T cells (Tregs), a T cell subset involved in preventing autoimmunity (101), is a critical step in immune development. The neonatal period is a key window for peripheral Treg induction, with specific microbial exposures during this window potentiating Treg development. For example, monocolonization of germ-free mice with *Bacteroides fragilis* increases Treg suppressive abilities and induces production of the immunomodulatory cytokine interleukin-10 (IL-10) from intestinal Foxp3+ Treg cells (102). Short-chain fatty acid production by the gut microbiome also promotes Treg development; butyrate and propionate both induce peripheral Tregs (103, 104). In mice, Treg induction depends on acquisition of an adult microbiota during weaning (105, 106). Because preterm infants have immature intestinal epithelia and aberrant microbiome development, this interplay is likely to be disrupted, perhaps contributing to diseases of immune dysregulation observed in preterm infants (Fig. 1C).

### IgA targeting of gut bacteria

IgA is the most abundant immunoglobulin in the intestine and is crucial for maintaining intestinal barrier function (107). Intestinal IgA production is stimulated by the microbiota and in turn regulates microbial abundance and association with the mucosa (107–110). Starting around the 4th week of life, sIgA is produced by B cells in the gut-associated lymphoid tissue (72, 111). This process is stimulated by the microbiome; germ-free mice produce negligible amounts of gut IgA (108). SIgA production is not equally stimulated by all gut microbiome members, with strain-specific differences in *Bacteroides ovatus* being a notable example of this phenomenon (44). In turn, sIgA increases the association of commensal bacteria, such as *Bacteroides* and *Lactobacillus,* with the mucosa (58, 107). SIgA also selectively depletes fast-growing bacteria from the microbiome by promoting their peristaltic elimination (112).

The result of this interplay is improved gut homeostasis and barrier function (113). Luminal sIgA prevents gut-to-lymph node translocation of potentially pathogenic bacteria, inhibits enterotoxin activity, impacts the developmental trajectory of the gut microbiome, and downregulates expression of pro-inflammatory genes in the intestinal epithelium (107, 110). Studies of murine NEC models have shown breast milk only protects against NEC when it contains sIgA, and breastfed pups that do not develop NEC have increased sIgA binding to NEC-inducing *Enterobacter* (64). Additionally, sIgA deficiencies in humans, especially lack of IgA in the gut, are associated with dysbiosis and systemic inflammatory disease (113–116). This indicates that sIgA is a key modulator of infant intestinal inflammation, highlighting the importance of developing therapeutic interventions to alter sIgA exposure or production to mitigate adverse outcomes.

## MICROBIOME DYSBIOSIS AND DISEASE STATES

### Sepsis

**Fig 2:**
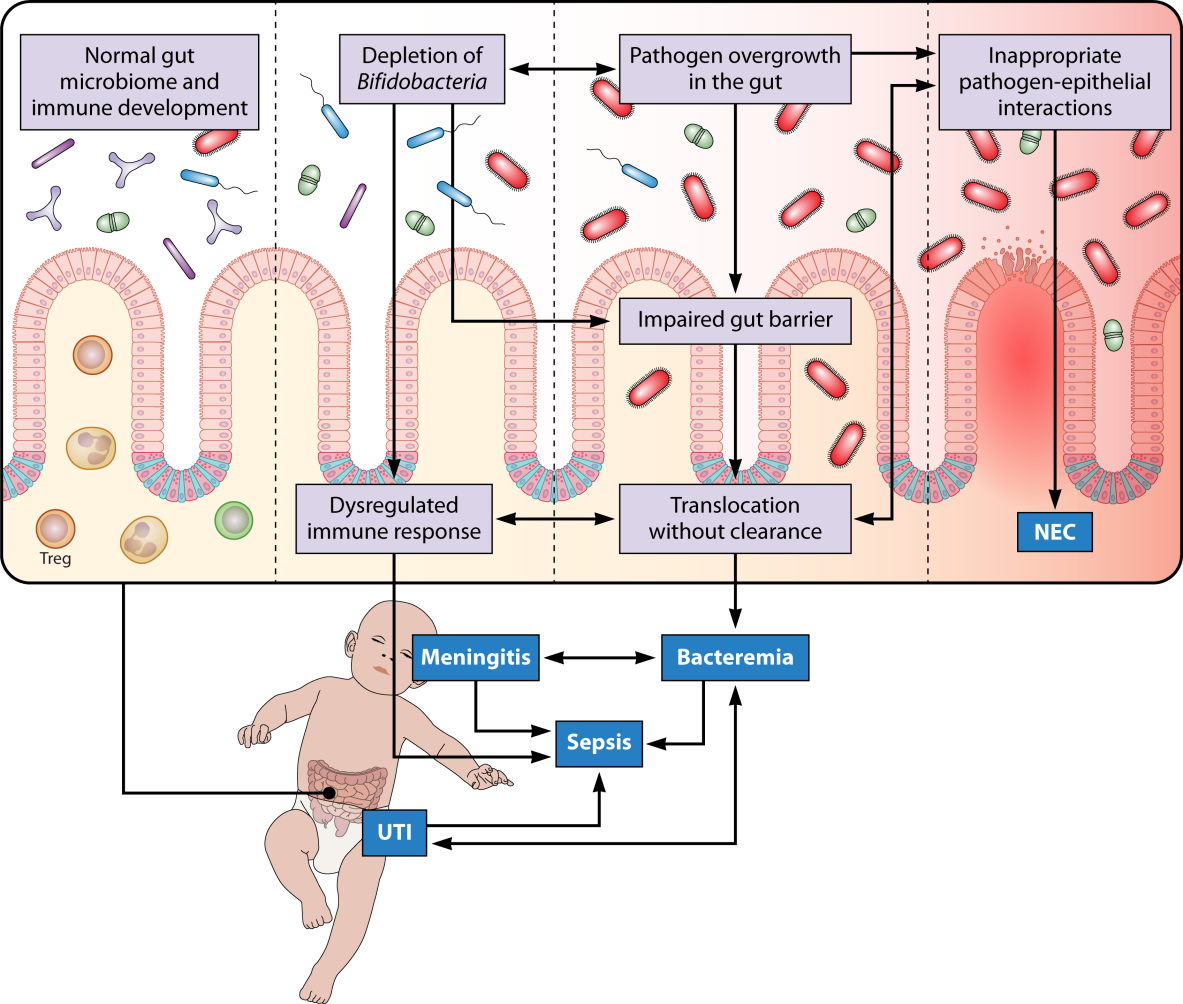
Microbiome disruption contributes to pathogenesis of serious bacterial infections, sepsis, and NEC. Depletion of *Bifidobacteria* and other beneficial anaerobes prevents normal immune and gut barrier development and reduces colonization resistance, allowing for pathogens to expand in the gut. This promotes bacterial translocation into the bloodstream. Bacteremia can lead to sepsis, meningitis, and UTI. Aberrant colonization and interactions with the epithelium underlie NEC pathogenesis.

Sepsis is a severe and maladaptive systemic immune response to suspected or confirmed infection that can result in organ failure, shock, and death (117). There is no consensus definition for neonatal sepsis, contributing to difficulty in diagnosis, clinical trials, and sepsis epidemiology (118). Hospitalized preterm infants are particularly vulnerable to sepsis and associated mortality (119, 120). Neonatal sepsis is most often precipitated by urinary tract infection (UTI), bacterial BSI, and meningitis (Fig. 2).

Empiric antibiotics are typically administered upon suspicion of sepsis, and diagnostics are taken to identify causative organisms, including bacteria, viruses, and fungi. If a bacterial pathogen is isolated from blood, urine, or cerebrospinal fluid (CSF), antibiotic treatment is tailored according to the identified organism and its antibiotic resistance profile. However, up to 49% of sepsis cases are culture-negative, meaning a causative agent cannot be identified (121). In culture-negative sepsis, clinicians are faced with a critically ill patient and must make important decisions regarding continuation of antibiotics. While in some cases, a decision to continue antibiotics could be life-saving, it can simultaneously cause harm. Prolonged use of unnecessary antibiotics is associated with increased risk for culture-positive sepsis, NEC, and death (122). Written guidelines regarding time-limited use for antibiotic durations can help standardize this clinical decision-making (123).

### Bloodstream infections

In the NICU, BSI is the most common precursor to sepsis (Fig. 2). Because of this, and the lack of consensus definition for neonatal sepsis, the terms sepsis and BSI are often used interchangeably. For consistency with the literature, this section uses sepsis and BSI synonymously. However, it is important to clarify that these are overlapping yet distinct entities: bacteria can be detected in the bloodstream without an accompanying inflammatory response, and sepsis can result from pathogens outside the bloodstream.

Neonatal BSI is classified by infant age at time of diagnosis. Sepsis occurring within the first 72 hours of life is classified as early-onset sepsis (EOS), while late-onset sepsis (LOS) occurs after this window. BSIs that cause EOS and LOS have distinct etiologies and clinical manifestations. EOS is typically caused by vertically transmitted pathogens from maternal fecal and vaginal microbiomes, such as Group B *Streptococcus* (GBS) and *E. coli* (124). In contrast, causative agents of LOS can be either vertically or horizontally transmitted (125). Among preterm infants, the top causative agents of gram-positive BSI/LOS are coagulase-negative *Staphylococci* (CoNS), *S. aureus*, and *Enterococcus* (126). CoNS and *S. aureus* are skin colonizers often involved with skin-to-bloodstream infections from catheters and other indwelling medical devices (127). They are also prevalent in preterm gut microbiomes (6, 29). The most common causes of gram-negative sepsis are *Enterobacteriaceae*, such as *E. coli*, *Klebsiella*, and *Enterobacter* (126). LOS risk is inversely correlated with GA (125, 128), with other significant risk factors including indwelling medical devices, prolonged NICU stay, empiric antibiotics, and parenteral nutrition (125, 129).

Substantial evidence indicates that the preterm gut microbiome is a reservoir for BSI-causing bacteria (30, 130–133). Bacterial strains and species colonizing the infant gut often match pathogens identified in blood cultures and extraintestinal infections (134–138). Recent work has demonstrated that the exact strain of BSI-causing bacteria exists in the gut microbiome and often increases in abundance before or coincident with sepsis onset (30), supporting gut-to-bloodstream translocation as a BSI pathomechanism (138). Notably, the presence of anaerobic commensals such as *Lactobacillus* and *Bifidobacterium* that provide colonization resistance against pathogens can protect against LOS (131, 137, 139).

Several studies have reported that microbiome disruptions precede BSI and sepsis (87, 140), and mouse models have provided mechanistic insights into how antibiotics and dysbiosis potentiate bacterial gut-to-bloodstream translocation. In neonatal mice treated with broad-spectrum antibiotics, *E. coli* and *Enterococcus* can be isolated from the liver and spleen (132). These mice exhibit reduced type 3 innate lymphoid cells (ILC3s) in the lamina propria and IL-17A production (132), which are both associated with protection against sepsis (141). Importantly, microbiome reconstitution restores ILC3s and reduces sepsis (132). Additionally, antibiotic-induced dysbiosis in neonatal mice impairs granulopoiesis and decreases neutrophil count, increasing susceptibility to *E. coli* and *K. pneumoniae* sepsis (141). Impaired clearance of translocating bacteria by neutrophils has been implicated in neonatal susceptibility to sepsis (131). Together, these data support the existence of a feedback loop between the gut microbiome, immune system, and sepsis development.

### Urinary tract infections

UTIs are the most common bacterial infections among preterm and term infants, affecting approximately 3% of febrile infants (142). Common causative agents of UTI in this age group include *E. coli*, *Klebsiella*, *Enterobacter*, *Enterococcus*, *Proteus mirabilis*, and *Pseudomonas aeruginosa* (143). Key risk factors include male sex, low GA, low birth weight, vaginal delivery, length of hospitalization, and circumcision status (144, 145).

Generally, UTIs arise as a primary infection, where bacteria colonize the periurethral area, invade the urethra, and ascend through the urinary tract. While some UTIs may occur secondary to bacteremia via hematogenous seeding of the urinary tract (Fig. 2), most studies examining blood and urine culture concordance have not definitively established the direction of this association (146–148). In ~10%–15% of cases, UTIs may progress to more serious conditions, such as pyelonephritis or BSI (146).

Gut microbiome perturbations have been linked to UTI development in adults and older children (149–151). Due to the anatomical proximity of the urethra and anus, periurethral contamination by gut bacteria is believed to be a common cause of UTIs, especially in diapered infants (152). However, the nature of this association appears to vary depending on patient characteristics (149, 153). Whether the infant gut microbiome serves as a reservoir for uropathogenic bacteria remains an open question, as does whether UTIs in this population result from gut-urethral migration of bacteria. To our knowledge, only four human cohort studies have examined gut microbiome dynamics in relation to UTIs in this age group. Two of these studies observed enrichment for uropathogens in the gut microbiomes of infants who later developed UTI with the same organism (154, 155). In the third, the causative uropathogens were either of low abundance or absent from the gut in UTI cases (156). Recently, we found increased *E. coli* abundance of an identical strain in the urine to the gut at ER presentation for febrile term infants versus controls without UTI (138). Further investigation is needed to confirm the generalizability of these findings and to evaluate potential use of the gut microbiome in predicting UTI risk in the NICU.

### Meningitis

Bacterial meningitis refers to inflammation of the protective membranes surrounding the brain and spinal cord with growth of a bacterial pathogen (157). In neonates, bacterial meningitis often precedes or complicates bacteremia (Fig. 2) (158). Meningitis is a more common manifestation of LOS than EOS and is associated with high mortality and severe neurological morbidities (158, 159). The epidemiology and etiology of bacterial meningitis in infants varies by postnatal age, age of onset, and geographic location. Incidence rates are likely underestimated, as patients often receive empiric antibiotics before a lumbar puncture is performed (157). In North America and Europe, the major causative agents of bacterial meningitis in neonates are GBS and *E. coli* (158, 160). Other important global causes among infants <3 months are *Acinetobacter baumannii*, *K. pneumoniae*, *Haemophilus influenzae,* and *Neisseria meningitidis* (161, 162).

The gut microbiome is thought to play an indirect role in neonatal meningitis by seeding BSIs. In mouse models, susceptibility to neonatal GBS meningitis has been linked to decreased colonization resistance from gut microbiome immaturity and incomplete polarization of cellular junctions in the intestinal and choroid plexus (163), which promotes bacterial translocation. To our knowledge, only one human cohort study has specifically examined the gut microbiome in neonatal meningitis. A study of preterm infants >30 days old found increased Proteobacteria and decreased Firmicutes and *Bacteroides* 1–10 days before meningitis onset using 16S rRNA sequencing (164). In half of the cases from this study, the causative agent’s genus was abundant in the gut microbiome, and 29% had a concurrent culture-proven BSI with the same genus. While these findings support the existing theory that neonatal meningitis can result from BSI caused by translocating gut bacteria, it remains unknown whether these microbiome changes are distinct from those associated with BSI. Higher-resolution sequencing and precise strain tracking are needed to clarify the gut microbiome’s role in neonatal meningitis.

### Necrotizing enterocolitis

NEC is a severe inflammatory disease of the intestinal lining, which can rapidly progress to ischemic necrosis, tissue sloughing, and intestinal perforation (165). NEC can be lethal and is primarily a disease of prematurity, with >90% of cases occurring in VLBW infants (166). Global incidence among VLBW infants in the NICU is approximately 7% (9). The etiology of NEC is not well understood and is likely multifactorial, but aberrant gut microbial colonization is recognized as a key feature of its pathogenesis (8). Early culture-based studies frequently implicated *Enterobacteriaceae* such as *E. coli*, *K. pneumoniae*, and *E. cloacae* (167). Animal models have identified Toll-like receptor 4 (TLR4), TLR9, and epidermal growth factor as host factors critical to pathogenesis (168–171), supporting the hypothesis that NEC results from inappropriate interactions between gut bacteria and the intestinal epithelium (Fig. 2). However, NEC-associated pathogens are also common in preterm infants who do not develop NEC. To date, no single microorganism has been identified as the universal cause of NEC.

Because of the concern for aberrant host-microbiome associations, NEC research has shifted toward examining patterns of intestinal dysbiosis associated with the condition. Earlier studies using 16S rRNA sequencing linked NEC to dominance or rapid expansion of γ-Proteobacteria (or its subfamily *Enterobacteriaceae*) and a decreased relative abundance of obligate anaerobes, such as Firmicutes and Clostridia-Negativicutes (172–175). However, these community-level alterations are common features among all hospitalized preterm infants, not just those who develop NEC (38). Recent shotgun metagenomic analyses associated the pre-onset NEC microbiome with significant *Klebsiella* enrichment and fimbriae-encoding bacteria, as well as increased bacterial replication rates (29, 176). Yet, other work suggests NEC is not a homogeneous process. Through multi-omics analysis of 48 NEC cases and 96 paired controls, the pre-onset NEC microbiome revealed no significant differences in bacterial composition, metatranscriptomic and functional profiles, virulence factor repertoires, or microbiota shift frequency (29). Notably, when NEC cases were subset to those experiencing NEC after 40 days of life, microbiome development stagnated, including an over-abundance of *Klebsiella* spp., suggesting a microbial trigger might exist for certain infants and reproducing earlier work in distinct cohorts (29, 176).

Collectively, existing metagenomic evidence supports the long-standing hypothesis that NEC results from pro-inflammatory interactions between potentially pathogenic bacteria, such as *Enterobacteriaceae*, and the underdeveloped preterm intestine. However, it remains unclear whether the inciting bacteria are aberrant strains colonizing the infant gut or persistent pathogens resulting from the failure of the preterm gut microbiome to diversify.

## DIAGNOSTICS AND AMELIORATION

### Judicious use of antibiotics

The increasing threat of multidrug-resistant pathogens has led to widespread implementation of antibiotic stewardship programs in clinical settings. However, in the NICU, empiric antibiotics are often initiated when infants exhibit clinical signs of infection while awaiting culture results from blood, urine, trachea, and CSF. If cultures are sterile, treatment may be continued, changed, or discontinued at the clinician’s discretion. Many clinicians choose to continue empiric antibiotics after sterile cultures due to the risk of false negatives and ongoing clinical decompensation (177–179). Although mortality rates are lower in cases of culture-negative sepsis compared to culture-positive sepsis (180), infants who receive prolonged courses of empiric antibiotics despite sterile cultures are at increased risk for NEC, LOS, and death (40, 181). Clinicians must weigh the risks of allowing missed infections to go untreated against the adverse effects of prolonged antibiotic use.

Therefore, there is a need for diagnostic tests with fast turnaround times and high sensitivity, enabling clinicians to discontinue or refine antibiotic use based on the identification of causative organisms. Some rapid blood culture diagnostic tests, including Verigene and Blood Culture Rapid Identification Panel, are FDA-approved and have contributed to antibiotic stewardship in BSIs (182, 183). Additional molecular methods to directly detect bloodborne pathogens are in development (184–187). Despite concerns regarding test sensitivity, specificity, and analyte volume requirements, these methods provide rapid results and may be particularly useful when combined with traditional blood cultures (184, 188). Meanwhile, examination of sepsis management and antibiotic use in the NICU illustrates that improvements in antibiotic stewardship are already achievable (189–191). For select groups, EOS risk can be stratified based on maternal factors and evolving clinical presentation (192, 193), and implementing a multivariable prediction model of EOS risk in late preterm and term infants reduced use of blood cultures and antibiotics during the first 72 hours after birth without increasing adverse outcomes (193).

### Gut microbiome surveillance

In the NICU, early detection of infections is critical to prevent infant mortality and morbidity. Because the infant gut microbiome serves as a reservoir for BSI-causing bacteria, we believe microbiome surveillance could inform risk assessment and allow clinicians to tailor antibiotic treatment based on its composition. In many LOS cases, the strain of bacteria responsible for BSI increases in the gut microbiota before infection (30). Additionally, microbiome composition could help triage potential causative agents of BSI, as microbes absent from the gut are unlikely to cause infection (30). For six species—*S. aureus*, *Streptococcus agalactiae*, *E. faecalis*, *Serratia marcescens*, *K. pneumoniae*, and *E. coli*—microbiome abundance thresholds showed >98% negative predictive value for sepsis caused by those organisms, which can help support the clinical decision to stop antibiotics (30).

Although sample-to-data turnaround time for metagenomic short-read sequencing is too long for this application, long-read sequencing technologies, such as Oxford Nanopore and PacBio, may be useful. Oxford Nanopore sequencing can produce long reads almost in real-time, enabling rapid pathogen identification, high-precision strain tracking, and ARG profiling (194). The MinION platform can be used to identify pathogenic bacteria and determine ARG profiles from infant stool samples within 5 hours of collection (194). As sequencing costs decrease, this is a promising approach to inform clinical decision-making.

### “-biotic” therapies

Significant efforts have been made to develop therapeutics targeting the gut microbiome to prevent or treat disease. Among these, probiotics and prebiotics have emerged as key yet contentious approaches.

Probiotics are living microbes administered to provide health benefits (195). Usage is not standardized across NICUs, resulting in variations in product formulations and clinical guidelines (196, 197). Common strains include *Bifidobacterium* spp.*, Streptococcus thermophilus*, *Lactobacillus*, and the yeast *Saccharomyces cerevisiae* var. *boulardii*. Meta-analyses suggest multi-strain probiotics containing *Bifidobacterium* and *Lactobacillus* spp. are promising for preventing NEC and reducing overall mortality (198–200). However, optimal strain combinations need to be identified, and evidence remains mixed (198–202). Current research indicates probiotics do not offer significant LOS prevention unless combined with lactoferrin (202–205).

Probiotics are not without risk; case reports have documented probiotic strains as causes of bacteremia in preterm neonates (206–209), and a systematic review of probiotic-related sepsis in this population identified 25 cases and two deaths (210). Although there is no evidence that probiotics increase overall odds of sepsis, the risk of bacteremia without clear data supporting efficacy has created hesitation to adopt them as a standard treatment (198, 200, 211). Furthermore, once probiotics are administered in a NICU, the probiotic strain can be detected in microbiomes of non-recipient infants and can persist in recipients’ microbiomes after treatment cessation (212). This raises concerns about the impact of standardizing probiotic administration in treatment facilities for vulnerable patients. At a minimum, probiotic strains should be carefully selected based on potential virulence factors, and host factors must be considered to ensure patient safety (208, 209).

Concerns regarding probiotic safety have driven the development of prebiotics—non-living substrates selectively metabolized by commensal bacteria for host benefit (213). Most prebiotics are synthetic or plant-derived carbohydrates such as inulin, galactooligosaccharides (GOS), fructooligosaccharides, and lactulose (214, 215). A 2023 meta-analysis of commonly used prebiotics in preterm and VLBW infants found little to no protective effect against NEC, all-cause mortality, or late-onset bacterial infections (215). Prebiotics may not be effective in preterm infants because their gut microbiome is often completely devoid of the commensal bacteria targeted by prebiotics. Synbiotics, which combine prebiotics and probiotics, seek to address this, although there is only low-certainty evidence regarding their efficacy (215).

Another emerging strategy is to recapitulate the benefits of breastfeeding by using HMOs as candidate prebiotics (213). In formula-fed term infants, supplementing formula with HMOs leads to a microbiome closer in composition to breastfed infants (216–218). Additionally, the HMO disialyllacto-N-tetraose is protective against NEC in rats, while GOS, a prebiotic currently added to infant formula, is not protective (219). Thus, although standard prebiotics have limited efficacy in preventing NEC, supplementing formula with HMOs may be a promising path forward.

### Microbiota transfers

Microbiota transfers aim to correct dysbiosis by transferring microbes from a “healthy” donor to an affected recipient. In contrast to probiotics, they contain intact microbial communities including bacteria, phages, and metabolites. In infants, microbiota transfers often aim to rescue disruptions to gut microbiome development caused by C-section. Vaginal microbiota seeding (VMS) and fecal microbiota transplant (FMT) are under investigation for this purpose.

VMS is conducted by swabbing C-section infants with vaginal fluid or by oral administration of vaginal fluid (220). VMS may enrich vaginal bacteria in neonatal microbiomes (221, 222) and shift C-section infant gut microbiomes closer to the gut microbiomes of vaginally delivered infants (221–223). However, other studies have shown that VMS results in nonsignificant changes to the gut microbiome and does not significantly impact allergy, growth, or other health outcomes (224, 225). Infant contact with maternal fecal material during vaginal birth, which VMS does not recapitulate, may facilitate colonization with important commensals such as *Bifidobacterium* and *Bacteroides*, which contributes to interest in FMTs.

While current FMT recommendations are largely limited to recurrent *Clostridioides difficile* infection, FMT is increasingly under investigation for the treatment of other gastrointestinal-related diseases (226–228). In infants, FMT is performed shortly after birth using an inoculum prepared from the infant’s mother’s fecal matter. A small clinical trial evaluated this procedure using orally delivered FMT to seven exclusively breastfed term infants (225). Infant FMT recipients had lower relative abundances of potential pathogens and higher abundances of commensal *Bifidobacterium* and *Bacteroides*, closely resembling vaginally born infants (225). These promising results have laid the foundation for an ongoing larger clinical trial assessing FMT in infants delivered at term via elective C-section (229).

Because the vagina and gut microbiomes can harbor pathogens, these approaches require thorough screening to ensure safety, and neither is currently allowed for use in infants outside of clinical trials (230, 231). Ultimately, it is unlikely microbiome transplants will be used for vulnerable preterm infants in the near future. More research is needed to establish standardized protocols for donor and pathogen screening, use cases, routes of administration, and dosing.

### Maternal vaccination

Maternal vaccines aim to prevent early-life infections by enhancing the protective capability of passively transferred maternal antibodies. Infants receive maternal antibodies by transplacental delivery of serum IgG and by ingestion of breast milk sIgA and IgG. Passively transferred antibody titers correlate with protection against neonatal sepsis and other early-life infections. Specific transplacental serum IgG is associated with a reduced risk of GBS (232) and *K. pneumoniae* neonatal sepsis (233), while milk IgA binding to *Enterobacteriaceae* in the infant gut protects against NEC (64).

A key limitation of maternal passive immunity is that the repertoire of transferred antibodies is shaped by the mother’s previous antigenic exposures (71, 234), which renders infants susceptible to pathogens if the mother does not produce sufficient antibodies against that pathogen. Maternal vaccines help overcome this limitation by inducing a specific anti-pathogen response in the mother, which is then transferred to the fetus or infant (235). Currently, Tdap is the only licensed antibacterial vaccine recommended for all pregnancies (236). However, several new maternal vaccines targeting bacterial agents are in development, including those for GBS (232, 237–239) and *E. coli* K1 (240). These vaccines primarily seek to transfer protective serum IgG responses, but in murine models, maternal vaccines can also stimulate milk sIgA responses that reduce intestinal colonization of bacterial pathogens in the infant gut (241).

The development of new maternal vaccines against bacterial pathogens is projected to significantly improve neonatal outcomes (242–244). For instance, a maternal *K. pneumoniae* vaccine could prevent an estimated 400,000 cases of infant sepsis and 80,000 deaths annually worldwide (243). Overall, maternal vaccines hold significant potential, and continued advancements in this field may lead to the inclusion of new antibacterial vaccines in standard prenatal care.

## CONCLUSIONS AND FUTURE DIRECTIONS

In preterm infants, stereotyped microbiome development is disrupted by a combination of host and environmental factors. This, along with immune disruption and immature organ systems, increases the risk of serious complications that can be traced to gut microbiome dysbiosis. While significant progress has been made investigating how gut microbiome-immune system interactions contribute to negative clinical outcomes in early life, much remains to be understood (245).

First, the understanding of the role of the preterm gut microbiome in sepsis, bacterial infections, and NEC is limited by a lack of clear, generalizable associations with specific microbiome features. Future studies should leverage large clinical cohorts to integrate high-resolution metagenomics, transcriptomic profiling of both the host and gut microbiome, and comprehensive environmental and clinical exposure data. This approach would improve confidence in the strength and generalizability of associations between dysbiosis, immune dysfunction, bacterial infections, and NEC (245). Gnotobiotic animal models and *in vitro* systems should be used to uncover specific host and/or microbial pathways involved in these interactions. Such information is essential to develop and improve risk prediction models and diagnostic algorithms, as well as to identify microbiome-based disease markers and inform strategies for treatment or prevention.

Second, due to the complexity and interconnectivity of the neonatal gut ecosystem, great care must be taken when applying microbiome-directed therapies in this vulnerable population. Approaches involving the direct administration of microbes, such as probiotics or microbial transfers, carry a significant risk of infection and introduction of potential pathogens into the NICU environment. Moreover, these interventions have not proven to be consistently effective against disease-associated dysbiosis of the preterm gut microbiota. However, alternative strategies that do not involve the direct administration of live microbes, such as promoting the ingestion of immunologic and prebiotic factors through breast milk, maternal vaccination, and improved antibiotic stewardship, show promise and warrant further investigation. Ultimately, these combined approaches may lead to improved diagnostics, treatments, and preventive strategies, resulting in better outcomes for preterm infants.
